# The Analysis of Patterns of Two COVID-19 Outbreak Clusters in China

**DOI:** 10.3390/ijerph19084876

**Published:** 2022-04-17

**Authors:** Wenning Li, Jianhua Gong, Jieping Zhou, Hongkui Fan, Cheng Qin, Yujiang Gong, Weidong Hu

**Affiliations:** 1 National Engineering Research Center for Geoinformatics, Aerospace Information Research Institute, Chinese Academy of Sciences, Beijing 100094, China; lwngreat@126.com (W.L.); zhoujp@aircas.ac.cn (J.Z.); qincheng20@mails.ucas.ac.cn (C.Q.); 2 University of Chinese Academy of Sciences, Beijing 100049, China; 3 School of Geology and Geomatics, Tianjin Cheng Jian University, Tianjin 300384, China; hongkuifan@126.com; 4 Zhejiang-CAS Application Center for Geoinformatics, Jiaxing 314100, China; iur2310@dingtalk.com (Y.G.); hwdrivermap@126.com (W.H.)

**Keywords:** COVID-19, China, system dynamics modeling, spatiotemporal evolution, outbreak in clusters

## Abstract

Since the emergence of COVID-19, there have been many local outbreaks with foci at shopping malls in China. We compared and analyzed the epidemiological and spatiotemporal characteristics of local COVID-19 outbreaks in two commercial locations, a department store building (DSB) in Baodi District, Tianjin, and the Xinfadi wholesale market (XFD) in Fengtai District, Beijing. The spread of the infection at different times was analyzed by the standard deviation elliptical method. The spatial transfer mode demonstrated that outbreaks started at the center of each commercial location and spread to the periphery. The number of cases and the distance from the central outbreak showed an inverse proportional logarithmic function shape. Most cases were distributed within a 10 km radius; infected individuals who lived far from the outbreak center were mainly infected by close-contact transmission at home or in the workplace. There was no efficient and rapid detection method at the time of the DSB outbreak; the main preventative measure was the timing of COVID-19 precautions. Emergency interventions (closing shopping malls and home isolation) were initiated five days before confirmation of the first case from the shopping center. In contrast, XFD closed after the first confirmed cases appeared, but those infected during this outbreak benefitted from efficient nucleic acid testing. Quick results and isolation of infected individuals were the main methods of epidemic control in this area. The difference in the COVID-19 epidemic patterns between the two shopping malls reflects the progress of Chinese technology in the prevention and control of COVID-19.

## 1. Introduction

The COVID-19 pandemic poses a global threat to human health and socioeconomics, and the impact of COVID-19 will continue for decades. Human-to-human transmission of COVID-19 has been confirmed in medical settings [1,2], wholesale markets [3,4,5], shopping malls [6,7,8], fitness centers [9], schools [10], and other public settings [11,12,13], and gathering in these public spaces has accelerated the spread of COVID-19. Without effective control measures, such as testing, isolation, personal protective equipment, and vaccines, viruses can spread. The spread of animals and plants, diseases, and new technologies are subject to a certain law of spatial interaction [14]. The spatial spread of epidemics follows the law of distance attenuation in geography and social physics [15]. The local gravity model based on power-law decay can effectively describe the diffusion patterns and process of COVID-19 in Hubei Province [16]. On a global scale, the risk of COVID-19 in each country decreased exponentially with the input of the first imported case and the time of onset [17]. At present, many types of COVID-19 vaccines are on the market. China is working at full throttle to vaccinate its people against COVID-19, and more than 3.2 billion doses had been administered as of 7 April 2022, according to the National Health Commission of the People’s Republic of China [18,19]. Studies have shown that vaccines are effective in preventing severe illnesses. However, considering issues such as vaccination rates and virus mutations, the effectiveness of vaccination-based prevention requires further assessment [20,21]. Moreover, social distancing remains one of the most effective means of controlling the spread of the virus. Therefore, it is important to study the characteristics of virus transmission via epidemiological simulation and assess the effectiveness of prevention and control measures in public places.

In China, government departments have researched infection control and tracing by collecting detailed case information and providing source data for studying spatiotemporal transmission. We compared and analyzed two outbreaks of COVID-19 in China and studied the characteristics of the spread from commercial centers. The first case was a local outbreak associated with a department store building in Baodi District, Tianjin, and the other case was an outbreak associated with the Xinfadi wholesale market in Fengtai District, Beijing. We performed the following research procedures based on a geographical analysis method: we established a spatiotemporal transmission model of the disease, created the spatiotemporal process map of the spread of the disease, extracted epidemiological information from the available situational data, and identified the similarities and differences in prevention and control measures during different periods in the two locations. In this study, we aimed to describe the epidemiological and spatiotemporal evolution characteristics of outbreaks in gathering places and the necessity of prevention and control methods. Our research provides a scientific reference for analyzing the key factors that contribute to local virus transmission and its prevention and control.

## 2. Materials and Methods

In this section, we mainly introduce the contents, preprocessing methods, and calculation methods of relevant epidemic indicators used for comparative analysis of the epidemiological data between the DSB and XFD outbreaks and for calculation of the reproduction number. We also introduce a method for measuring the spatiotemporal distributions of epidemics that was used to compare the change trends of the two outbreaks in space and time. In addition to presenting relevant epidemic prevention and control strategies, we propose an improved dynamic model for evaluation of the importance of nucleic acid detection and early epidemic tracing in epidemic prevention and control.

### 2.1. Data Sources

**Figure 1:**
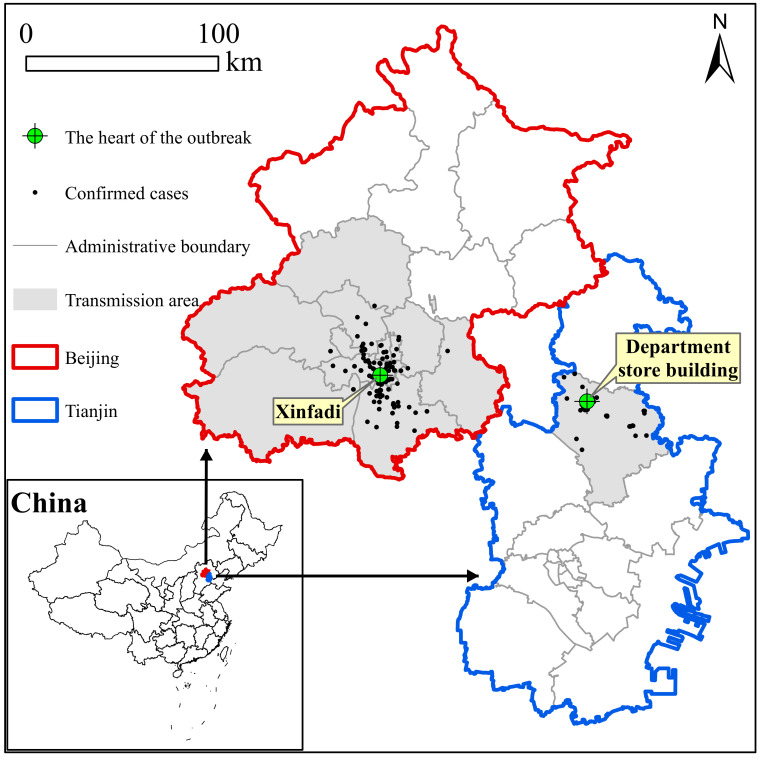
Location and case distribution of two outbreaks in market clusters in Tianjin and Beijing.

From the *Health Commission of Tianjin* (http://wsjk.tj.gov.cn, accessed on 1 March 2020), the data for confirmed cases related to DSB were collected. The notification time range of confirmed cases was from 31 January 2020 to 21 February 2020, and there were 60 confirmed cases in this epidemic period [8]. The database contained detailed anonymous information on confirmed cases (including sex, age, workplace, activity tracing, date of symptoms, confirmed date of diagnosis, case transmission relationship, and residence location). Additionally, confirmed case data related to the XFD epidemic were obtained from the *Beijing Centers for Disease Prevention and Control* (https://www.bjcdc.org, accessed on 1 August 2020). The time range of confirmed and reported cases was from 11 June 2020 to 5 July 2020, with a total of 335 confirmed cases. The data included sex, age, residence location, workplace, date of symptoms, date of diagnosis, and clinical classification. The distribution of cases from the two epicenters is shown in Figure 1. As this study constituted data analysis rather than human research, ethical approval from institutional review boards was not required.

### 2.2. Estimation of the Effective Reproduction Number

The effective or net reproduction number on day t (Rt) is an important indicator for monitoring and tracking the progress of epidemics. Rt represents the average number of secondary infections per initial infection case [22,23,24]. After the implementation of the corresponding control measures, the transmission intensity of the virus will immediately change accordingly. However, data regarding the response to these measures (e.g., the number of confirmed cases) will be delayed; the temporal component of Rt monitoring can negate the effect of this delay. Therefore, compared with the number of newly confirmed cases reported, Rt estimation can better reflect changes in the transmission intensity. The calculation of Rt is based on the method and software developed by Abbott et al. [25].

### 2.3. Spatiotemporal Analysis

To analyze the spatial and temporal spread of the epidemics and measure the development direction and distribution range of the epidemics at different times, we used the standard deviation ellipse (SDE) method (Supplementary Text S1) [26,27]. The SDE approach quantitatively describes the spatial distribution characteristics of a given factor, such as the corresponding centrality, directionality, and spatial form, through a spatial distribution ellipse with the center, major axis, minor axis, and angle of rotation as the basic parameters. The elliptical spatial distribution range describes the main area of the spatial distribution of geographical elements. The center describes the relative position of geographical features in two-dimensional space. The azimuth describes the main trend direction of the geographical element distribution. The long axis represents the degree of dispersion of geographical elements in the trend direction [28]. We geolocated the residential locations of confirmed cases in a GIS-based map using ArcGIS 10.2 (ESRI, Redlands, CA, USA). Combined with the SDE method, the disease outbreak in each period was illustrated to intuitively display the characteristics of disease transmission.

### 2.4. Modified Dynamic Model

We used the modified SEIR model to fit the spread of the virus and simulate the sensitivity of different intervention measures. The model is shown in Figure S1. The model mainly consists of four statuses: susceptible, exposed, infectious, and reported. According to the changes in the exposure rate and the nucleic acid testing rate after the implementation of different policies, the number of COVID-19 infections was simulated. It should be noted that the model assumes that confirmed cases are highly isolated and have negligible transmission capacity. As some parameters in the model cannot be measured directly, we used the Monte Carlo method to reconstruct the virus propagation growth curve from historical data and known parameter values. R^2^ (the coefficient of determination) was used as a standard to measure the prediction results of the model. Additionally, we analyzed the effects of the isolation tracking time and comprehensive nucleic acid testing on virus transmission.

## 3. Results

### 3.1. General Characteristics

The DSB outbreak included clusters with 60 confirmed cases. It took 23 days for all related cases to be confirmed, and they included 25 males (41.67%) and 35 females (58.33%). The median age of infected individuals was 50 years (IQR 36–64) (Table 1). Among all cases, 29 individuals had direct contact with the DSB (48.3%, 6 employees, and 23 customers). More than half of the individuals (51.7%, 31 cases) were infected outside the shopping mall; that is, they had no travel or shopping history from 20 January to 26 January 2020 (the closing date of the DSB). We calculated a median latency of 9.5 days (range 3–19, IQR 6–12). The median duration from symptom onset to reporting was 6 days (range 0–26, IQR 3–9).

There were 335 confirmed cases in the XFD outbreak. All relevant cases were found and confirmed within 25 days. Among all cases, 187 individuals were male, accounting for 55.82% of the total number of infected individuals, and 148 cases were female, accounting for 44.18% of all infected individuals; the median age of the infected was 43 (IQR 31–52). There were 246 cases (73.4%) of direct contact with the XFD. More than half of the infected individuals worked in the market, and approximately one-quarter of the cases were close contacts of infected persons, contaminated goods, or the contaminated environment. Among them, 94 individuals were able to pinpoint the exact exposure time. The latency was calculated as 5 days (IQR 3–8), and the median duration from symptom onset to reporting was 2 days (IQR 1–4).

**Figure 2:**
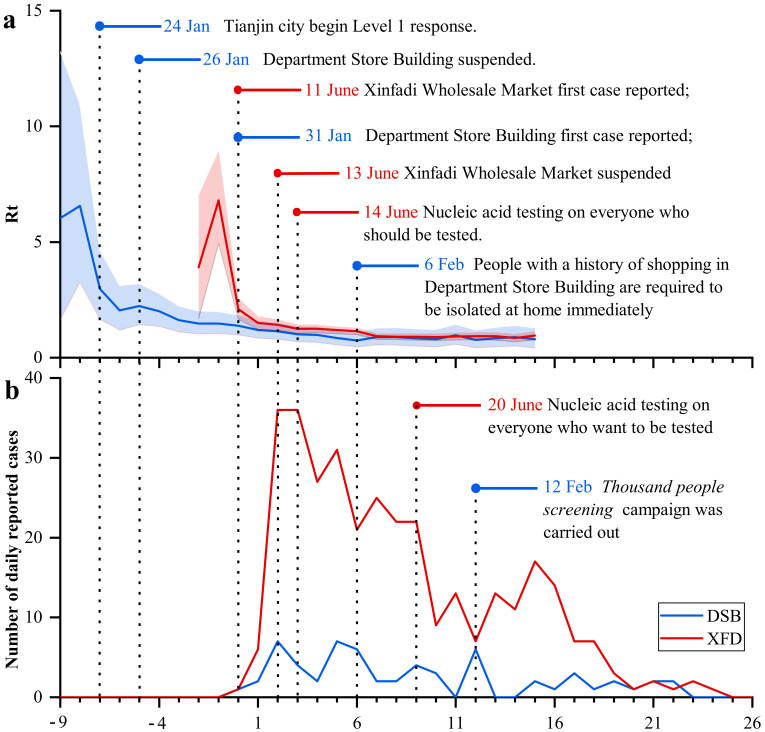
Prevention and control measures: (**a**) Rt; (**b**) number of daily reported cases. The *x*-axis indicates the number of days from the reported date of the first case. DSB-related information is represented in blue; XFD-related information is shown in red.

To quickly control the spread of COVID-19, the local governments in the two outbreak areas implemented various public health measures, as shown in Figure 2. During the period from the first virus carrier input to 26 January (when stores closed), the DSB region was in the early stage of the Chinese Spring Festival. At this time, many customers visited densely populated shopping malls, and Rt was estimated to reach a maximum of 6.56. The first associated case was confirmed on 31 January, and then 194 department store employees were sent to the centralized observation center for isolation on 1 February. The DSB outbreak was strictly managed. At this time, the Rt value decreased to less than 1. A series of close contact tracing and isolation measures were implemented, resulting in the complete control of the epidemic. On 6 February, the COVID-19 Command and Control headquarters in Baodi District issued a request that people who had a history of shopping in department stores immediately stay at home. On 7 February, all villages and communities were closed. On 9 February, three lines of defense were established at the district, urban, and village levels, and the 92,000 residents of the region were isolated at home. On 12 February, the novel coronavirus pneumonia epidemic protocol in the Baodi District of Tianjin was launched, followed by the “Ten Thousand People” screening campaign.

At the beginning of the XFD outbreak, Rt was as high as 6.8. On 12 June, the day after the first case was diagnosed, XFD was identified as the source of the infection. Nucleic acid screening for market personnel and individuals in the surrounding environment was initiated immediately. On 13 June, the XFD was closed. The surrounding communities were also closed and disinfected, and Rt dropped sharply to a level of 3.0. On 14 June, the authorities began testing all employees of the XFD and residents living in medium- and high-risk areas. The intervention was called “*Nucleic acid testing for everyone who should be tested*”. On 16 June, Beijing authorities lowered the emergency response to the public health alert level from 3 to 2. Rt maintained a downward trend and finally fell below 1 on 18 June, two weeks after the first case occurred. Beginning on 20 June, anyone desiring a NAT could go to a medical institution and be tested. This strategy was called “*Nucleic acid testing for everyone who wants to be tested*”.

### 3.2. Spatiotemporal Distributions

**Figure 3:**
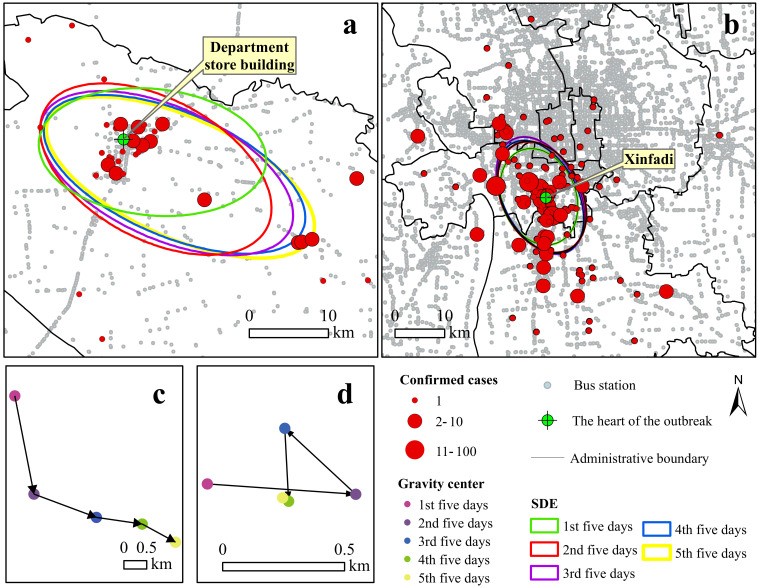
Spatiotemporal distribution of cases: (**a**,**b**) the distribution range and diffusion direction of confirmed cases of DSB and XFD in five time periods, respectively; (**c**,**d**) the change in direction of the ellipse center of DSB and XFD, respectively.

We extracted the geographical coordinates of the confirmed cases by geocoding and plotted them on the map. In cases for which the specific location could not be determined, the address of the nearest district administrative office was used instead. The spatial distributions for the DSB and XFD cases are shown in Figure 3. Spatially, the DSB epidemic was concentrated in and distributed across 34 districts and administrative villages, and approximately half of the confirmed cases were in 21 urban districts (33 cases, 55%). Over time, the virus spread from the central area to rural areas in the southeast, and the edge of the spread was dominated by family aggregations. The maximum straight-line distance of the spread was 35.13 km (Shuangzhuang village, Bamencheng town). All cases were controlled within Baodi District. The spatial distribution of XFD cases indicates that the spread was mainly concentrated in urban areas rather than rural areas (Figure 3), involving 112 communities and blocks in 11 districts of Beijing. Fengtai District and Daxing District were the areas most affected, accounting for 88% (295) of the cases. In Fengtai District, most cases were in Huaxiang town. Over time, the virus spread from the central area to the northwest and southeast. The farthest straight-line distance of the epidemic spread was 37.19 km to Tongzhou District.

We divided the epidemic process into five 5-day time periods. The standard deviation ellipse of each time period was calculated, and the resulting parameters are shown in Table S1. A comparison of the elliptical major axes revealed that the major DSB axis was larger than that for XFD, indicating that the DSB outbreak spread over a greater distance. The comparison of ellipse center indicators indicated that the center of the DSB shifted mainly to the southeast, as shown in Figure 3c. In contrast, the center of the XFD exhibited a small offset and moved only 1 km, as shown in Figure 3d, which indicated that the virus transmission was mostly contained in the outbreak epicenter. Overall, the XFD situation was better controlled than the DSB situation.

### 3.3. Sensitivity Analysis of Different Control Measures

**Figure 4:**
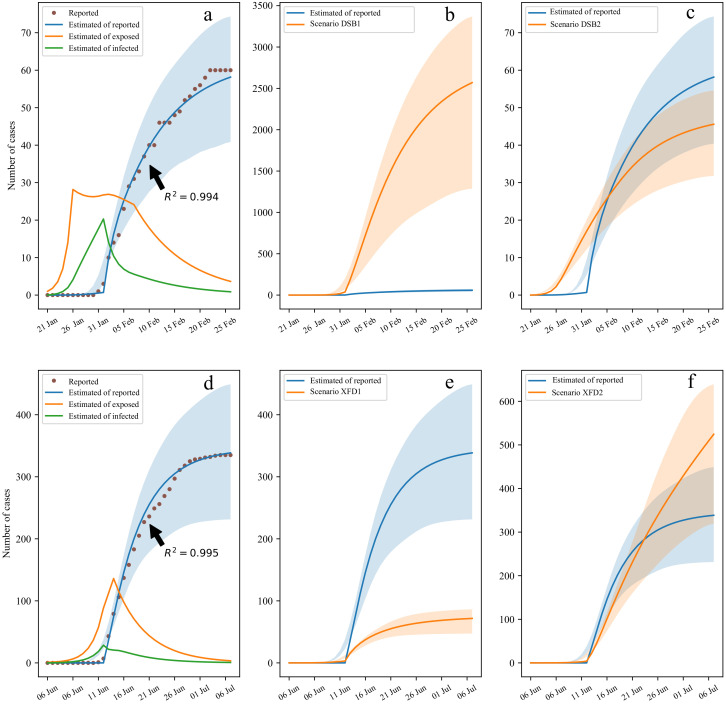
Sensitivity analysis of control measures based on the improved SEIR model: (**a**–**c**) simulations of the number of people diagnosed in DSB; (**d**–**f**) simulations of the number of people diagnosed in XFD.

We fitted the daily cumulative number of confirmed DSB and XFD cases. The R2 values simulated by the improved SEIR model were 0.994 and 0.995, respectively, as shown in Figure 4a–d. We compared the prevention and control measures of the two outbreaks in clusters in reference to the time of lockdown and comprehensive nucleic acid testing. We simulated the spread of the epidemics under different scenarios to verify the sensitivity of the two measures in the model. The DSB epidemic scenarios were as follows: In scenario DSB1, local blockading and isolation measures were implemented 5 days later than the actual situation (i.e., the time of the first case). It was predicted that there would be more than 2000 cases (95% CI 1307–3429 cases) in Baodi District on 25 February, 42.8 times the actual number reported, as shown in Figure 4b. In scenario DSB2, large-area nucleic acid testing was performed, and the final infection case number was 45 (95% CI 32–55), or 15 fewer infections than actually reported, as shown in Figure 4c. The XFD epidemic scenarios were as follows: In scenario XFD1, the epidemic was discovered 5 days after the actual situation; public places were shut down, and individuals were isolated. These measures resulted in 72 cases (95% CI 47–87 cases) on 6 July, 4.7 times fewer cases than actually reported, as shown in Figure 4e. In scenario XFD2, the nucleic acid testing rate was reduced by half, with 539 cases (95% CI 331–667 cases) on 6 July, which was 1.6 times higher than the actual number reported, as shown in Figure 4f. In this case, the epidemic situation was detected as soon as possible and controlled and isolated, which provided time for epidemic prevention and control in the DSB outbreak. This result supported the launching of the first-class public health response nationwide. Large-scale nucleic acid testing in the XFD outbreak area quickly led to the identification of infected individuals and shortened the range of spread and the time required to locate all infected individuals, thereby requiring minimal social isolation. In general, we believe that regular sampling nucleic acid testing is an effective means for detecting epidemics as soon as possible. Cui et al. similarly analyzed the XFD outbreak and concluded that prompt identification of the Xinfadi wholesale market as the infection source had an important impact on the number of people infected with XFD; they also believed that nucleic acid testing had the most significant effect [29].

## 4. Discussion

Our study did not describe the infection status of COVID-19 cases in detail but analyzed the effects of the similarities and differences in epidemic prevention and control in the two locations at different points in time. We evaluated the prevention and control results by comparing the temporal and spatial diffusion modes and prevention and control measures for the two aggregated epidemic cases. In addition, we conducted a comparative discussion of the characteristics of basic case information, epidemiological characteristics, and spatial transmission characteristics to explore the factors related to COVID-19 transmission in densely populated commercial areas and to provide scientific evidence for prevention and control policies for outbreaks in clusters.

The distance between DSB and XFD is approximately 83 km. According to epidemiological investigation and transmission chain analysis [29,30], the outbreak epicenters both support commercial activities (different from schools, high-speed railway carriages, and airports). The infected individuals were mostly employees and shoppers in both cases, allowing for an effective analysis of the two outbreaks. Notably, based on the case information for the two COVID-19 outbreaks, the total number of XFD cases was significantly higher than that for DSB, partly due to the population flow and floor area of XFD, which are larger than those of department stores in Baodi District. The XFD is the largest wholesale market in Asia, covering an area of approximately 1.12 million m^2^, and there are approximately 2000 permanent employees in the market. The DSB covers an area of approximately 3400 m^2^ and has approximately 200 employees.

The epidemiological information of the two outbreaks was compared. In terms of the age of infected persons, XFD cases were younger, with a median age of 43 years (IQR 31–52), while DSB cases had a median age of 49.5 years (IQR 37–63). In terms of the sex of infected persons, more women than men were infected in the DSB outbreak (the ratio of males to females was 5:7), and more men than women were infected in the XFD outbreak (187:148). This phenomenon is related to the type of shopping malls in the two districts. DSB is an urban retail center with more female customers. XFD is a large wholesale mall, with predominately male staff. There was no obvious sex difference in the spread of COVID-19. In terms of the diagnosis and treatment of cases, in XFD the time interval from the onset of symptoms to diagnosis was 2 days (IQR 1–4), which was significantly less than the 6 days (IQR 3–9) in the DSB epidemic. This is due to the rapid diagnostic abilities and sufficient medical resources in the region, as well as the public’s awareness and cooperation in epidemic prevention and control. In addition, some studies on COVID-19 pathology found that more consolidated lung lesions appeared five days after the onset of the disease, and the lesions were the most serious within 10 days. Therefore, rapid medical treatment is of great significance to the spread of the disease but also better outcomes for patients. In the XFD epidemic, severe patients accounted for only 0.896% of all cases.

In mapping the distributions for the DSB and XFD cases, we found that the two groups had similar spatial distribution patterns: (1) the virus spread from the central area to the periphery, the density of infected persons on the periphery was low, and most of the cases were the result of clusters at home or work; (2) the longest distances of viral spread in the two locations were similar (approximately 30 km), and there was no long-distance cross-city transmission, as shown in Figure S2a,b; (3) the area within 10 km of the epicenters was the most affected, and the number of infections there accounted for 65% and 82% of the total number of infections in the DSB and XFD, respectively; additionally, the number of infected individuals plotted as a logarithmic curve and displayed a negative correlation with increasing distance, as shown in Figure S3a,b. In terms of the form of transmission, there were 11 secondary transmission outbreaks in clusters related to the DSB, and 51.7% of the cases of infection occurred outside the mall. In contrast, 73.4% of XFD cases were the result of direct contact in the market. From the perspective of diffusion, the prevention and control were better for XFD than for DSB. In view of these findings, we compared the epidemics to outbreaks in chess and card rooms in Yangzhou on 28 July 2021, and found similar effects, as shown in Figure S3c. Notably, (1) there were no new confirmed infections 30 days after the implementation of control measures; (2) a 10 km radius from the epicenter was the main infection area (98%); (3) in all cases, the farthest distance from the epicenter was 28.8 km (all cases were within approximately 30 km); (4) most of the infected people in peripheral areas were secondary transmissions as a result of family or work clusters.

The reasons for the above differences between the two epidemic situations are reflected in the prompt identification of the epidemic source and extensive nucleic acid testing (Figure 4). The DSB incident occurred during the initial outbreak of COVID-19 in China. Medical technology and prevention and control measures were not fully in place at that time; notably, rapid and effective detection methods were lacking, and people’s awareness of COVID-19 prevention and control measures was poor. Thus, an important reason for the rapid control of the DSB epidemic was that the first-level public health response was launched nationwide and closed the DSB five days before the discovery of the first case. The government’s mandatory social isolation measures played a key role in the prevention and control of virus spread at this location. During the later XFD outbreak, virus detection and prevention, and control measures were relatively mature. Many people had some awareness of recommended precautions, such as disinfection in public places, wearing masks, and social distancing, and had read relevant medical information. After the first XFD case was confirmed, the source of the disease was determined on the same day, and blocking, isolation, and close contact tracing were immediately performed. A series of measures, such as “home quarantine”, “nucleic acid testing on everyone who should be tested”, and “nucleic acid testing on everyone who wants to be tested”, was implemented, and the number of confirmed patients was controlled at 335.

## 5. Conclusions

The frequent outbreaks of COVID-19 in shopping malls highlight the enormous challenge of pandemic control. Through a comparative study, we identified some key factors leading to virus transmission. People who work in crowded places such as shopping malls are often in contact with a polluted environment, and the infection rate there is high. Occupational health guidelines or legislation for COVID-19 prevention and control by employers in gathering places should be strengthened. Furthermore, there may be insufficient overall awareness of the disease in rural areas and insufficient attention to the severity and harm of the disease. This gap is evidenced by people who did not change their habits after the outbreak and continued to gather and share meals, which led to many clustered outbreaks in families. A large market-related epidemic is a major public health event. If appropriate control measures are not taken, it may lead to a pandemic such as COVID-19, and the key to solving this problem is to target the market-exposed population. NAT and big data contact tracing are effective methods that need to be implemented. Targeted interventions should also be deployed, including local blockades, close-contact tracing, and community-based testing, which have proven effective in controlling the spread of infection.

In this paper, we focused on the spatiotemporal transmission and diffusion characteristics of viruses, but the study has some limitations due to the SEIR model applied. The model did not consider the rehabilitation and removal of infected persons and assumed that the confirmed isolated cases no longer have the opportunity or ability to spread the virus while ignoring the supply of medical resources and beds; therefore, the simulation results can only be used in sensitivity assessments of prevention and control measures. In future research, more influencing factors will be comprehensively considered to improve the applicability of the model.

## Figures and Tables

**Table 1:** Characteristics of DSB and XFD outbreaks in clusters.

Characteristics	DSB	XFD
Outbreak time		
Notification time of the first confirmed case	31 Jan 2020	11 Jun 2020
Notification time of the last confirmed case	22 Feb 2020	5 Jul 2020
Time taken to detect all associated cases	23 days	25 days
Demographic Characteristics		
Confirmed	60	335
Male	25 (41.7%)	187 (55.8%)
Female	35 (58.3%)	148 (44.2%)
Age (median (IQR ^1^))	50 (36~64)	43 (31~52)
Incubation period: median (range)	9.5 (6~12)	5 (3~8)
Days from onset to confirmation: median (IQR ^1^)	6 (3~9)	2 (1~4)
Prevention and control measures		
Market suspension	Y	Y
Home quarantine	Y	Y
Close contact tracing	Y	Y
Nucleic acid testing	Y	N
Vaccination	N	N
Lockdown	Y	N
Death status		
Nondeath case	59	335
Death case	1	0
CFR (%)	1.7	0

^1^ IQR: interquartile range; CFR: case fatality rate.

## Data Availability

The data used to support the findings of this study are available from the authors upon request.

## Supplementary Materials

The following supporting information can be downloaded at: https://www.mdpi.com/article/10.3390/ijerph19084876/s1, Text S1. The standard deviation ellipse (SDE) method, Figure S1: Improved SEIR model, Figure S2: Distribution of infected persons at different distances from each epidemic center, Figure S3: Relationship between distance and cumulative number of confirmed patients, Table S1: SDE parameters.
