# Supplementary material for: The Analysis of Patterns of Two COVID-19 Outbreak Clusters in China
Source: Int J Environ Res Public Health. 2022 Apr 17;19(8):4876. doi: 10.3390/ijerph19084876 (PMC9032159; doi:10.3390/ijerph19084876)
Supplement: Supplementary file 1 [file ijerph-19-04876-s001.zip › ijerph-1664678-supplementary.pdf]

Text S1. The standard deviation ellipse (SDE) method

The calculation method is mainly divided into three steps: 1. Determine the center of the circle. 2. Determine the rotation angle. 3. Determine the length of the major and minor axes of the ellipse. The specific formula is as follows:

$$SDE_x = \sqrt{\frac{\sum_{i=1}^{n}(x_i - \bar{x})^2}{n}}, SDE_y = \sqrt{\frac{\sum_{i=1}^{n}(y_i - \bar{y})^2}{n}}$$

where (x_i, y_i) are the coordinates of the place of residence of a confirmed infected individual (called a confirmed case), $(\bar{X}, \bar{Y})$ represents the mean center of a case, and n is equal to the number of confirmed cases.

The angle of rotation is calculated as follows:

$$\tan\theta = \frac{A + B}{C}$$

$$A = \left(\sum_{i=1}^{n}\tilde{x}_i^2 - \sum_{i=1}^{n}\tilde{y}_i^2\right), \mathrm{B} = \sqrt{\left(\sum_{i=1}^{n}\tilde{x}_i^2 - \sum_{i=1}^{n}\tilde{y}_i^2\right)^2 + 4\left(\sum_{i=1}^{n}\tilde{x}_i\tilde{y}_i\right)^2}, C = 2\sum_{i=1}^{n}\tilde{x}_i\tilde{y}_i$$

where $\tilde{x}_i$and $\tilde{y}_i$ are the deviations of (x, y) from $(\bar{X}, \bar{Y})$.

The standard deviations for the x-axis and y-axis are as follows:

$$\sigma_x = \sqrt{2}\sqrt{\frac{\sum_{i=1}^{n}(\tilde{x}_i\cos\theta - \tilde{y}_i\sin\theta)^2}{n}}$$

$$\sigma_y = \sqrt{2}\sqrt{\frac{\sum_{i=1}^{n}(\tilde{x}_i\sin\theta - \tilde{y}_i\cos\theta)^2}{n}}$$

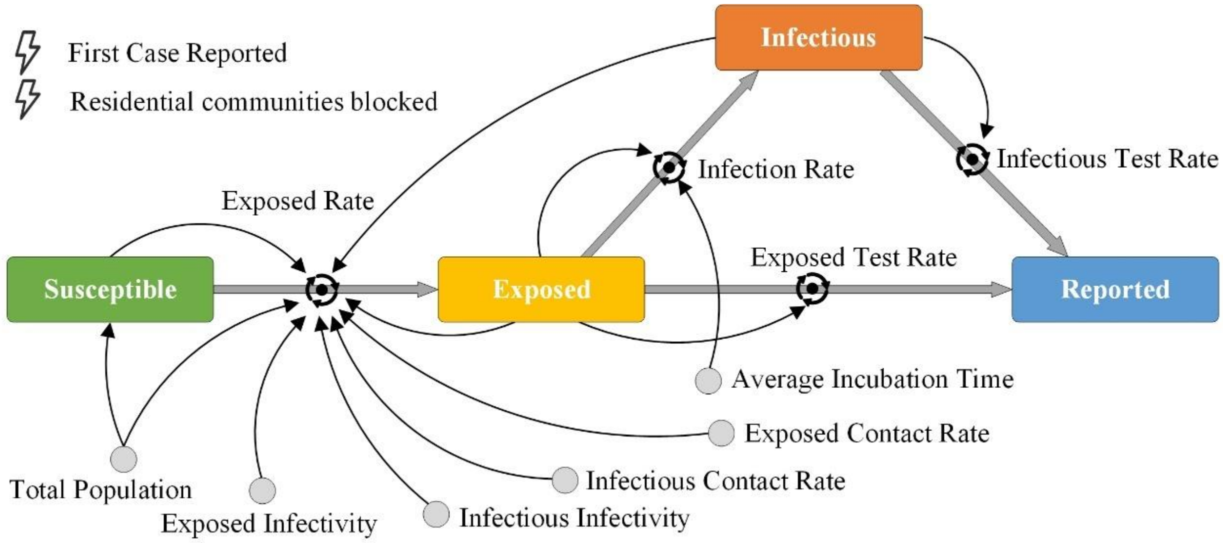

Figure S1. Improved SEIR model: *Susceptible* indicates people who have not been infected with the virus; *Exposed* indicates that the person infected with the virus has no symptoms but has a certain level of infection; *Infectious* means a person has symptoms of infection and can infect others; and *Reported* indicates a confirmed case. *Total population* refers to the total population of the study area; *Exposed rate* indicates the flow of susceptible people into exposed areas; *Exposed infection* refers to the probability that an exposed person is in contact with an easily infected person; *Infectious infectivity* refers to the probability that an infectious person contacts a susceptible person and the susceptible person is infected; *Exposed contact rate* indicates the average daily contact rate between an exposed individual and others; *Infectious contact rate* indicates the average daily contact rate between infectious individuals and others; *Average incubation time* indicates the number of days that the infection incubation period will last after a susceptible person is infected; *Infection rate* indicates the number of people exposed to the number of people infected; *Exposed test rate* the number of exposed individuals that tested positive; *Infection test rate* indicates the number of infected individuals confirmed through testing; ϟ *First case reported* refers to the first confirmed event; and ϟ *Residential communities*

blocked indicates where the outbreak center was closed. The contact rate and detection rate will change after two events.

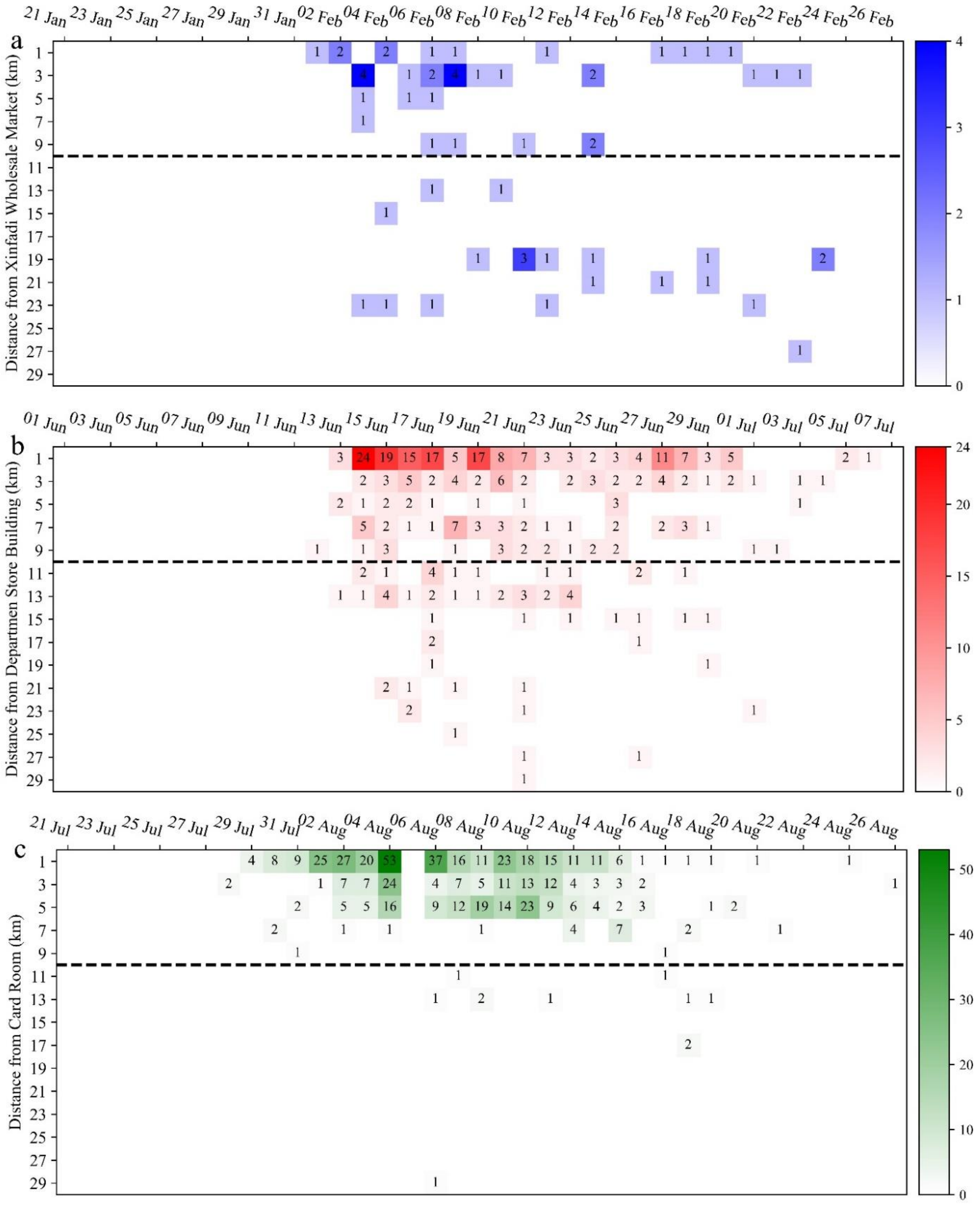

Figure S2. Distribution of infected persons at different distances from each epidemic center: (a) DSB; (b) XFD; and (c) Yangzhou, China.

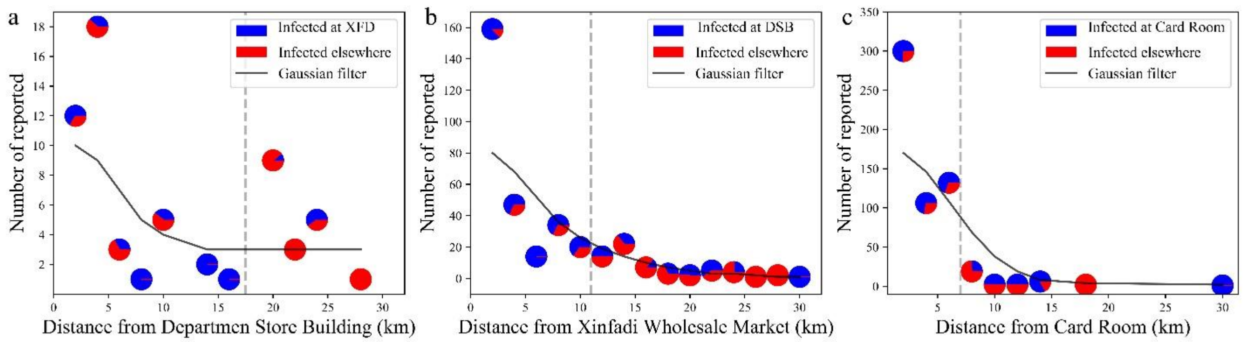

Figure S3. Relationship between distance and cumulative number of confirmed patients. (a) DSB; (b) XFD; (c) and Yangzhou, China. In the distance range on the left side of the dotted line, most pie charts are blue, indicating that the infected persons in these closed areas had a history of travel to and from the outbreak epicenter and were initially infected there. In contrast, the areas on the right side of the dotted line indicate secondary transmissions.

Table S1. SDE parameters.

	Time（5-day interval）	Mean center of the ellipse	Major axis	Minor axis	Angle of rotation (°)
DSB	1	117.339685, 39.71346	14410.97031	7983.848072	95.660481
	2	117.343448, 39.698568	15881.69875	8862.051235	118.180586
	3	117.355738, 39.695046	17151.44528	8286.043083	114.487726
	4	117.364755, 39.694042	17654.07863	7832.475908	113.225958
	5	117.371308, 39.691293	18248.09318	7579.023963	114.158147
XFD	1	116.327475, 39.820445	10012.19084	7440.223231	158.26746
	2	116.332955, 39.820063	11872.18671	8013.049218	153.090592
	3	116.330333, 39.822501	12552.15259	7743.995365	153.657724
	4	116.330474, 39.81981	12292.68242	7493.913747	152.770441
	5	116.330254, 39.819936	12188.9407	7438.226395	152.65551
